# Diagnostic Value of MRI Lamellated Hyperintense Synovitis in Periprosthetic Infection of Hip

**DOI:** 10.1111/os.12789

**Published:** 2020-11-22

**Authors:** Zongyan Gao, Yi Jin, Xiao Chen, Zhipeng Dai, Shuo Qiang, Shuyuan Guan, Qiang Li, Jincheng Huang, Jia Zheng

**Affiliations:** ^1^ Department of Orthopedics People's Hospital of Zhengzhou University, Henan Provincial People's Hospital, People's Hospital of Henan University Zhengzhou China; ^2^ Department of Medical Imaging People's Hospital of Zhengzhou University, Henan Provincial People's Hospital, People's Hospital of Henan University Zhengzhou China

**Keywords:** lamellated hyperintense synovitis, MRI, periprosthetic infection of joint, total hip arthroplasty

## Abstract

**Objective:**

To investigate the correlation between magnetic resonance imaging (MRI) lamellated hyperintense synovitis and periprosthetic infection of hip arthroplasty and estimate its value in the diagnosis of infection after hip replacement.

**Methods:**

A retrospective analysis of 50 patients who underwent MRI from January 2016 to June 2019 after hip replacement was performed. The MRI scanning was performed with a 1.5T clinical imaging unit using SEMAC protocols. A total of 25 patients (cohort 1) showed infected total hip arthroplasty, and 25 patients had non‐infected arthroplasty as controls (cohort 2). Two musculoskeletal radiologists, blinded to the clinical diagnosis, reviewed all the images for the presence of lamellated hyperintense synovitis independently. The cases were rereviewed by each reader after 2 weeks. The sensitivity, specificity, positive predictive value, and negative predictive value were calculated using the first reads. The Kappa statistic was used to assess inter‐observer and intra‐observer reliability.

**Results:**

The incidence of lamellated hyperintense synovitis was 76%–88% in the experimental group and 8%–16% in the control group. The sensitivity of lamellated hyperintense synovitis for infection was 0.80–0.88 (95% confidence interval [CI]:0.59 – 0.97), the specificity was 0.84~0.92 (95% CI: 0.64  –0.99), the positive predictive value 0.83–0.92 (95% CI: 0.67 – 0.98), the negative predictive value 0.81 – 0.88 (95% CI: 0.65 – 0.96). The agreement between two readers was substantial (Kappa = 0.76, 95% CI: 0.58 – 0.94, *P* < 0.05). There were moderate inter–observer agreements for both readers, reader 1 (Kappa = 0.48, 95%CI: 0.23 – 0.72, *P* < 0.05) and reader 2 (Kappa = 0.44，95% CI: 0.19 – 0.69, *P* < 0.05).

**Conclusion:**

In this cohort, the presence of lamellated hyperintense synovitis in the MRI of hip arthroplasty showed high sensitivity and specificity for infection. This sign had substantial intra‐observer reliability and moderate inter‐observer reliability in the classification of the synovial pattern.

## Graphical abstract

The arrows show MRI LHS in PJI

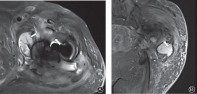

## Introduction

Total hip arthroplasty (THA) is the most extensively employed artificial joint replacement surgery^1^, ^2^. Periprosthetic joint infection (PJI) is one of the most challenging complications after THA^3^. According to the statistics of Swedish joint replacement registration, PJI is the third leading cause of hip revision surgery^4^. Owing to its prolonged course, it causes significant psychological and economic burden to patients and society. The early diagnosis of PJI is one of the key factors that determine the therapeutic effect. The current common treatment options include debridement lavage and second‐stage replacement based on antibiotic treatment. The former is suitable for patients with symptoms for <3 weeks or within 1 month after the surgery, and the prosthesis is well fixed. The treatment success rate of retaining the original prosthesis is 80%–93%. Typically, patients beyond this duration present the formation of biofilms and can only be treated by two‐stage renovation^5^.

Presently, the most commonly used diagnostic standard of PJI is the consensus of the International Conference on Periprosthetic Infection proposed by the American Society of musculoskeletal infection in 2013 (International Consensus Meeting 2013 [ICM 2013])^6^. It is mainly evaluated in terms of symptoms, physical signs, laboratory examination, the culture of puncture fluid, and pathology. This standard is helpful for early diagnosis but is limited for the evaluation of joint parts such as infection range, prosthesis, and the surrounding tissue. Conventional imaging examination, such as X‐ray and computed tomography (CT) imaging, is not included in the diagnosis standard because of the lack of resolution of soft tissue^7^. At present, MRI is the preferred non‐invasive imaging method for soft tissue resolution. However, conventional MRI scanning sequence used in the examination of metal endophyte cases produces artifacts and the imaging quality is poor, which limits its application. In recent years, the development of the technique with respect to the removal of metal artifacts of MRI has improved the quality of the images containing metal endophytes. Thus, due to its advantages of tissue contrast, it can be used for the evaluation of various THA postoperative complications, including PJI^8^, ^9^, ^10^.

Lamellated hyperintense synovitis (LHS) in an MRI image refers to the thickened synovial tissue around the joint in MRI, and “high signal” refers to the high signal in T2WI sequence image. This image was first found to be characteristic in the knee infection cases, but not in other inflammatory diseases, such as rheumatoid arthritis and osteoarthritis^11^. The study by Plodkowski *et al*. showed that the metal‐removing artifact MRI technology could be applied to patients with discomfort after total knee arthroplasty (TKA); the sensitivity of LHS to PJI diagnosis after TKA can reach 92% and the specificity can reach 87%^11^. Li *et al*.^12^ demonstrated that the examination of the patients with discomfort after TKA operation with the removal of metal artifact MRI identifies the synovitis caused by infection, wear particles, and nonspecific (pain, stiffness, or instability) among which LHS is the characteristic manifestation of infection. However, the diagnostic value of PJI after THA is currently lacking.

This retrospective study analyzed the images of THA postoperative cases using MRI scans with metal‐free artifacts and explored the diagnostic value of LHS for PJI after THA.

## Materials and Methods

### *Inclusion and Exclusion Criteria*

This study was approved by the Ethics Committee of Henan Provincial People's Hospital.

### *Inclusion Criteria*

The inclusion criteria were as follows: (i) participants – patients with periprosthetic hip infection after THA; (ii) intervention – patients who underwent 1.5T MRI dementalization sequence examination, and the final diagnosis was based on the diagnostic criteria proposed by the international consensus conference on periprosthetic infection in 2013^13^; (iii) comparison – patients with and without lamellated hyperintense synovitis; (iv) outcome – incidence of LHS; and (v) study design – retrospective cases study.

#### *Exclusion Criteria*

The exclusion criteria were as follows: (i) patients with periprosthetic infection and periprosthetic fracture or other complications simultaneously; (ii) the quality of MRI was not sufficient to evaluate the soft tissue around hip joint; and (iii) patients who cannot be diagnosed without a complete treatment process.

### *General Information*

According to the above inclusion and exclusion criteria, we reviewed 72 patients who underwent MRI examination for different reasons after THA in our department from January 2016 to June 2019, and 64 patients exhibited a clear diagnosis. Among these patients, 25 who had been diagnosed with PJI after THA and underwent the operation and follow‐up antibiotic treatment were included in the infection group (group 1), and 25 patients were randomly selected from other diagnoses to form the control group (group 2). The age, gender, interval time from THA operation to MRI examination, and the final diagnosis were recorded.

### *Study Methods*

The 1.5T MRI scanner (Siemens, Germany) has the following advantages^8^: (i) the scanner's 1.5T field strength combined with SEMAC sequence can suppress the metal artifacts; (ii) it is safe for patients with metal implants. The specific imaging parameters are listed in Table 1.

**TABLE 1:** MRI imaging sequence

Scanning parameters	Axial positionT1WI‐SEMAC	Coronal T1WI‐SEMAC	Axial position T2WI‐tirm‐SEMAC	Coronal T2WI‐tirm‐SEMAC
TR (ms)	475	471	5000	3000
TE (ms)	9.5	9.5	61	56
Layer thickness/layer spacing (mm)	4/0	4/0	4/0	4/0
FOV (mm × mm)	220 × 220	220 × 220	220 × 220	260 × 260
Matrix	175 × 320	189 × 320	162 × 256	196 × 320
Bandwidth (Hz)	488	488	488	488
Acceleration factor	2	2	2	2
Parallel acquisition factor	2	2	2	2
Echo chain	27	59	12	18
Flip angle (°)	150	139	138	150

The images were independently interpreted and recorded by two professional musculoskeletal radiologists, blinded to the final clinical diagnosis of the case. Physicians No. 1 and No. 2 have 10 years and 8 years of MRI diagnostic experience, respectively, and each has 3 years of periprosthetic MRI diagnosis experience.

### *Outcome Evaluation*

#### *LHS*

**Fig 1:**
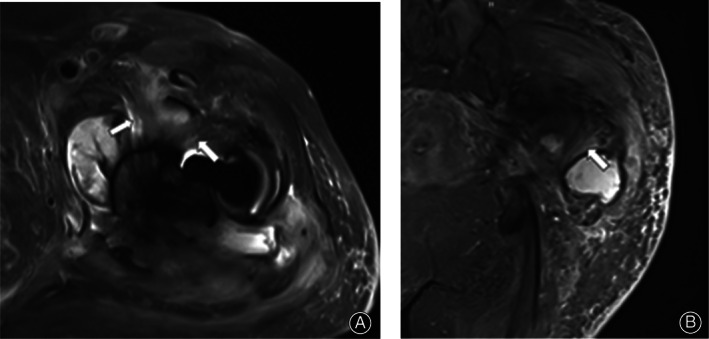
A 73‐year‐old female patient underwent THA for 4 years. On T2WI‐tirm‐SEMAC, the synovium around the joint in the axial position (A) and coronal position (B) showed LHS (arrow), which could be distinguished from the normal muscle and tendon tissues by anatomical tissue location, travel, and morphology. The patient has a history of diabetes and complained of resting pain in the right hip for >4 months. Admission examination revealed an erythrocyte sedimentation rate of 80 mm/h, C‐reactive protein was 71.81 mg/L, and white blood cells were 8.3 × 10^9^/L. Surgery was performed; a large amount of purulent effusion was found during the operation, and the number of neutrophils was >5/high magnification field of vision. The final diagnosis was PJI.

**Fig 2:**
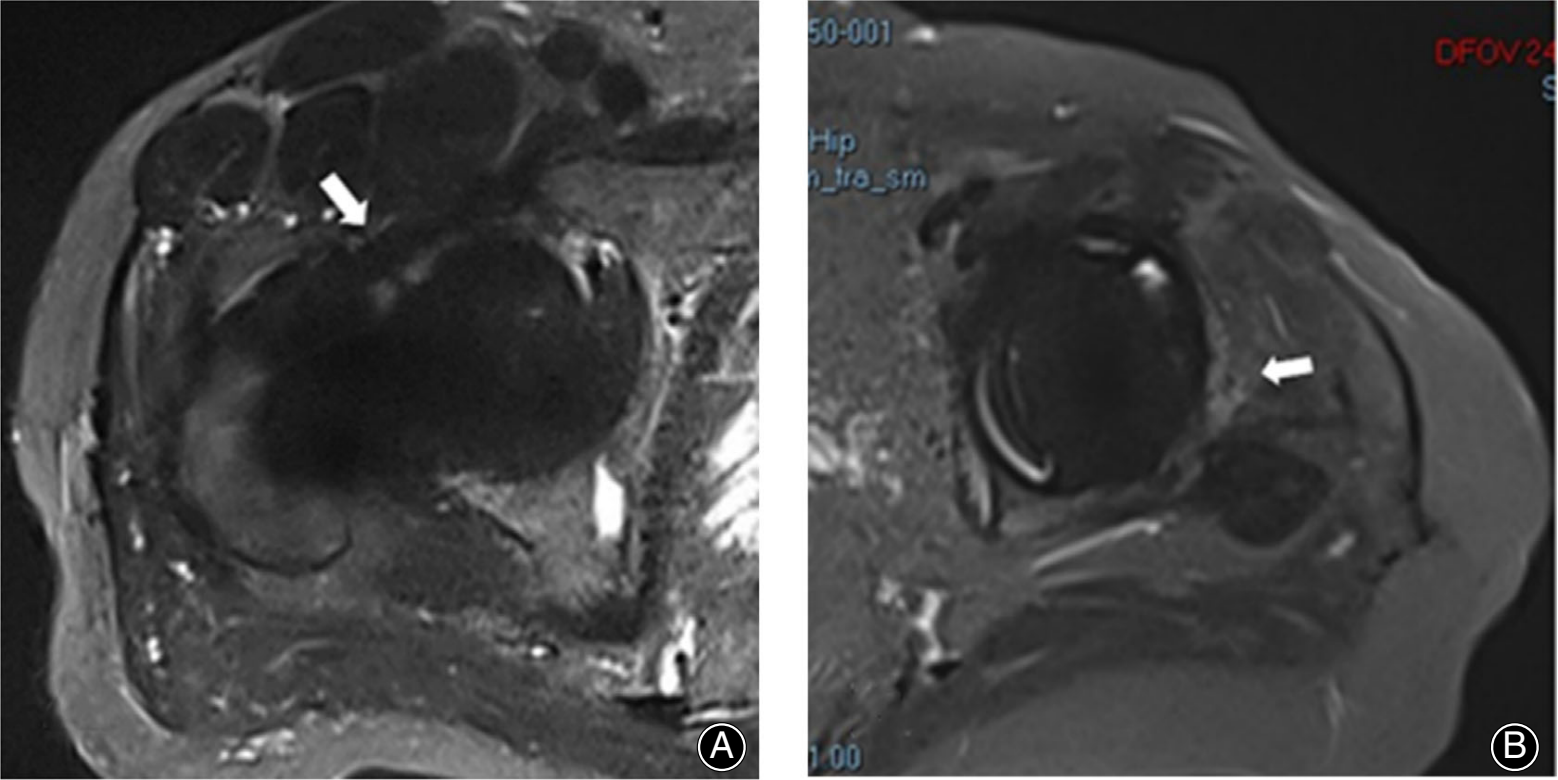
T2WI‐tirm‐SEMAC axial images of two patients underwent THA after MRI examination. (A) Male, 48‐years‐old, 1 year before THA, the postoperative recovery was better without complaints, and the image was obtained at the time of reexamination. A clear articular cavity boundary was observed in front of the joint, and no LHS image was obtained in the axial position of the joint (arrow). (B) Female, 63‐years‐old, 11 years before THA surgery, hip pain for half a year as the main complaint at the time of admission, diagnosis of aseptic loosening of the prosthesis after THA surgery received revision surgery, arrow indicates light high signal soft tissue shadow, but does not have the lamellated characteristics.

The phenomenon is defined as a lamellated thickening of synovium tissue around the joint, “high signal” refers to high signal on T2WI‐tirm‐SEMAC image, as shown in Fig. 1. Figure 2 shows the MRI image of non‐infected patients, which was interpreted as no LHS. The film reading is only determined in the presence of LHS.

#### *Consistency Test*

Two radiologists read the film twice at an interval of 2 weeks. The results of the first film reading were used to calculate the sensitivity, specificity, positive predictive value, negative predictive value, and intra‐observer reliability of LHS.

#### *Repeatability Test*

After 2 weeks, the film was read again, and the results were compared with the previous results of the same observer to evaluate the observer's self‐stability.

### *Statistical Analysis*

Kappa consistency analysis was used to analyze the reliability among observers^14^ at 95% confidence intervals (CIs). The classification definition of consistency was as follows: Kappa coefficient 0.81–0.99 was extremely excellent, 0.61–0.80 was excellent, 0.41–0.60 was good, 0.21–0.40 was medium, 0.01–0.20 was poor. All *P*‐values were set to α <0.05. The differences between the infection and control groups in terms of gender, age, and interval time from THA operation to MRI examination were used for the χ^2^ test, *t* test, and nonparametric test, and *P* < 0.05 was considered statistically significant. All statistical analyses were performed using MedCalc statistical software (MedCalc, version 18.2.1, Belgium).

## Results

### *General Information*

Group 1 (infection group) consisted of 25 patients. The duration from THA to MRI examination was 1–204 (average: 51.7) months. All patients received follow‐up surgery and antibiotic treatment, including 22 cases of exclusion and three cases of tube irrigation. Group 2 (control group) consisted of 25 patients. The duration from THA to MRI examination was 3–216 (average: 85.0) months. Among them, 16 cases of aseptic loosening, six cases of asymptomatic reexamination, one case of periprosthetic fracture, and two cases of pain syndrome with significant pain relief were detected after conservative treatment. No significant difference was observed in the gender and age between the two groups, but the time from THA to MRI examination differed significantly between the two groups (*P* = 0.037).

### *Consistency Test*

The incidence of LHS with two film readings by two observers in the experimental group was 76%–88%. The statistical analysis results of the reading results of the two radiologists are shown in Table 2. Furthermore, the consistency between the two radiologists was excellent (Kappa = 0.76, 95% CI: 0.58–0.94, *P* < 0.05).

**TABLE 2:** General situation of infected and control groups

	Case	Male	Female	Age (years)	Time * (months)	Diagnosis
Group 1	25	14	11	60.0 ± 13.6	51.7 ± 50.7	PJI
Group 2	25	12	13	65.0 ± 13.2	85.0 ± 61.1	Aseptic loosening 16 cases periprosthetic fractures 1 case Pain syndrome 2 cases Stable and asymptomatic prosthesis 6 cases

^*^ Time from THA operation to this MRI examination.

PJI, periprosthetic joint infection

### *Repeatability Test*

**Fig 3:**
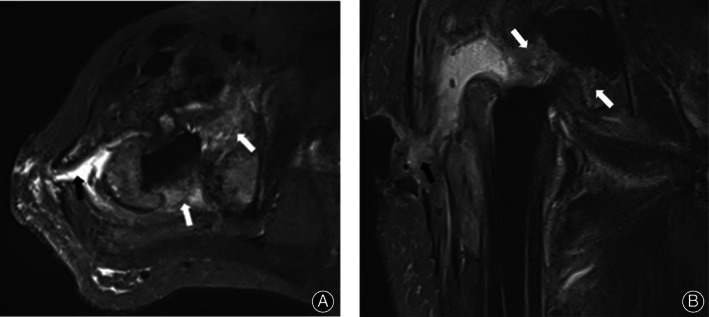
A 62‐year‐old male patient underwent THA for 1 month, and the incision healed poorly after the operation. MRI examination was performed at 1 month after surgery. T2WI‐tirm‐SEMAC axial position (A) and coronal position (B) showed edema in the joint cavity and surrounding tissues with mixed‐like high signals (white arrows) and formed sinus tracts that reached the body surface (black arrow). The patient was diagnosed with PJI in parallel with catheter irrigation and subsequent antibiotic treatment. Due to the mixed characteristics of the patient's synovium and surrounding soft tissues, both radiologists interpreted it as negative for LHS.

The self‐consistency of the two radiologists was good: radiologist 1 (Kappa = 0.48, 95% CI: 0.23–0.72, *P* < 0.05), radiologist 2 (Kappa = 0.44, 95% CI: 0.19–0.69, *P* < 0.05). In the infection group, three patients were judged to have no LHS by two radiologists at the first reading (Table 3). MRI was performed in two cases of infection within 2 months after the operation, and the image of one case is shown in Fig. 3.

**TABLE 3:** statistical analysis results of LHS as MRI indication of periprosthetic infection

	Radiologist 1	Radiologist 2
Sensitivity	0.80 (0.59–0.93)	0.88 (0.69–0.97)
Specificity	0.84 (0.64–0.95)	0.92 (0.74–0.99)
Positive predictive value	0.83 (0.67–0.93)	0.92 (0.74–0.98)
Negative predictive value	0.81 (0.65–0.90)	0.88 (0.72–0.96)

95% CIs in parentheses.

LHS, lamellated hyperintense synovitis

## Discussion

### *Development of* *PJI* *Diagnosis and Radiology*

Currently, the most important indexes of PJI diagnosis are the bacterial culture of the sinuses, the joint puncture, and the results of histopathology around the joint. Other auxiliary indexes include erythrocyte sedimentation rate (ESR), C reactive protein, and joint fluid cell count^13^, ^15^. X ray and CT are used for the detection of soft tissue and CT also displays interference of prosthesis metal artifacts; thus, the sensitivity and specificity of PJI diagnosis needs to be improved. MRI has the advantages of high popularity, good tissue comparison, and noninvasiveness. In addition, the application of MRI in PJI diagnosis was also limited due to the metal artifacts. In recent years, MRI studies have found that the metal artifact produced by 1.5T field strength scanning is smaller than 3.0T field strength^8^; on the other hand, the application of de‐metallic artifact sequences has greatly improved the imaging quality and has been successfully utilized for the evaluation of complications after joint replacement^8^, ^16^. In the early stage of PJI, the soft tissues around the prosthesis are involved, deeming MRI conducive for imaging analysis. In this study, we used Siemens 1.5T MRI scanner and SEMAC sequence to remove the metal artifacts, and cooperated with the engineers to optimize the scan parameters, which suppressed the metal artifacts and obtained high‐quality imaging.

### *Diagnostic Value of* *LHS* *to* *PJI*

Fritz *et al*. described the characteristics of MRI of complications after hip replacement through retrospective analysis and literature summary. Among these, subcutaneous sinus tract, abscess, bone marrow edema, and lymphadenopathy suggested the existence of PJI^8^. LHS was first found as a characteristic MRI of periarticular abscess^11^. Due to the increased application of MRI for periprosthetic scanning, Plodkowski *et al*. found that LHS also has an optimal diagnostic value and reliability for PJI cases after TKA^11^. However, the diagnostic value of PJI after THA is yet lacking. In this study, the diagnostic value of PJI after THA was evaluated by a retrospective control study.

The results showed that LHS had better sensitivity, specificity, positive predictive value, negative predictive value, and consistency among the observers for PJI, which supported that LHS had an accurate diagnostic value for PJI after THA. Furthermore, the abundant soft tissue that was detected around the hip joint was rich, but the characteristics differed from those of the knee joint. Based on Fig. 3 as an example, although a diffuse mixed high signal could be seen around the joint, it lacks lamellated characteristics; hence, both radiologists interpreted it as negative for LHS, indicating that although LHS has a good diagnostic value, using a single indication to determine infection might cause reproducibility deficiencies. According to the pathological characteristics of infection, the accuracy of diagnosis can be further improved if the integrated sinus formation and extensive tissue are mixed with high signal. In the present study, a significant difference was detected in the gender and age between the infection and control groups, but the time from THA to this MRI examination was longer in the control group than in the infection group. According to the incidence rate of postoperative complications of THA, the first is aseptic loosening^4^; thus, the proportion of aseptic loosening in the control group was the largest and was primarily related to wear particles and osteolysis at the interface of the prosthesis, rendering a long onset of aseptic loosening after the initial operation. In addition to blood‐borne infections, most of PJI occurs within 2 years after THA. Also, while reviewing the cases with long intervals in the infection group, LHS was detected. Therefore, we speculated that LHS is not related to the interval between THA and MRI examination but is mainly affected by the characteristics of the disease.

Nevertheless, the present study has two limitations. First, the sample size is small, and the number of cases with complete diagnosis and treatment in the past 3 years is limited. This is also consistent with the incidence rate of PJI 1%–2%^6^. In the future, the research reliability can be improved, and better image diagnosis standards can be formulated by multicenter joint research. Second, reportedly, LHS rarely occurs in inflammatory arthritis other than infection and complications after joint replacement^11^, ^12^. This retrospective analysis lacked the histological basis. Thus, we speculated that this phenomenon is related to chronic inflammatory edema and cell infiltration and proliferation caused by infection.

### *Conclusion*

In the MRI examination, the removal of metal artifact sequence scans provided an optimal sensitivity, specificity, and positive and negative predictive value of LHS for the diagnosis of PJI after THA with improved diagnostic value.
