# Correction to “Diagnostic Value of MRI Lamellated Hyperintense Synovitis in Periprosthetic Infection of Hip”

**DOI:** 10.1111/os.14154

**Published:** 2024-07-22

Gao, Z. , Jin, Y. , Chen, X. , Dai, Z. , Qiang, S. , Guan, S. , Li, Q. , Huang, J. and Zheng, J. (2020), Diagnostic value of MRI lamellated hyperintense synovitis in periprosthetic infection of hip. Orthop Surg, 12: 1941–1946. 10.1111/os.12789 33225607 PMC7767676

On page 1941, the second sentence in the Result under Abstract section is incorrect. The sentence should be as follows:

There were moderate intra‐observer agreements for both readers, reader 1 (Kappa = 0.48, 95% CI: 0.23–0.72, *p* < 0.05) and reader 2 (Kappa = 0.44, 95% CI: 0.19–0.69, *p* < 0.05).

Table citations in the Results and Discussion section were amended. Furthermore, reference citations 16 to 20 were added in the Discussion section.

In the General Information under Result section, the last sentence should be read as “No significant difference was observed in the gender and age between the two groups, but the time from THA to MRI examination differed significantly between the two groups (*p* = 0.037) (Table 2).”

In the Consistency Test under Result section, the second sentence should be read as “The statistical analysis results of the reading results of the two radiologists are shown in Table 3.”

In the Repeatability Test under Result section, the second section should be read as “In the infection group, three patients were judged to have no LHS by two radiologists at the first reading (Table 4).”

**TABLE 4:** Patients in each group determined to have LHS by the radiologists.

	Radiologist 1		Radiologist 2	
	1st time	2nd time	1st time	2nd time
Group 1	20 (80%)	19 (76%)	22 (88%)	19 (76%)
Group 2	4 (16%)	2 (8%)	2 (8%)	3 (12%)

In the Development of PJI Diagnosis and Radiology under Discussion section, it should be corrected as follows:

Currently, the most commonly used parameters, according to the international consensus conference on periprosthetic infection,^13,15^ include C reactive protein (CRP) and erythrocyte sedimentation rate (ESR) in serum, synovial white blood cell (WBC) count and polymorphonuclear neutrophil% (PMN%), and intraoperative histology. These parameters have a variety of diagnostic values and limitations (Table 5). In comparison with LHS, synovial WBC count and PMN%^16^ have lower positive predictive values (76.4% and 59.3%, respectively), synovial culture^17^ and Intraoperative histology^18^ have lower sensitivity (55% and 73.7%, respectively), and serum CRP and ESR have lower specificity^19^ (78.3% and 65.1%, respectively). Radiology, which has the advantages of being noninvasive and rapid, plays an important role in disease diagnosis. However, conventional radiological techniques, such as X‐ray and CT, perform poorly in diagnosing PJI due to the lack of soft‐tissue resolution and metallic artifacts in CT images. MRI has the advantages of high popularity, good tissue comparison, and noninvasiveness. In addition, the application of MRI in PJI diagnosis was also limited due to the metal arti‐facts. In recent years, MRI studies have found that the metal artifact produced by 1.5T field strength scanning is smaller than 3.0T field strength^8^; on the other hand, the application of de‐metallic artifact sequences has greatly improved the imaging quality and has been successfully utilized for the evaluation of complications after joint replacement.^8,20^ In the early stage of PJI, the soft tissues around the prosthesis are involved, deeming MRI conducive for imaging analysis. In this study, we used Siemens 1.5T MRI scanner and SEMAC sequence to remove the metal artifacts, and cooperated with the engineers to optimize the scan parameters, which suppressed the metal artifacts and obtained high‐quality imaging.

**TABLE 5:** Diagnostic values and limitations of different methods for PJI.

	Sensitivity	Specificity	PPV	NPV	Limitations
Aspiration					Limited PPV, Invasiveness, difficulty in aspirating the hip
WBC count^16^	89.5%	91.2%	76.4%	97.5%	
PMN%^16^	92.1%	85.8%	59.3%	98.0%	
Culture^17^	55.0%	100.0%	100.0%	61.0%	Limited sensitivity, invasiveness, prolonged incubation
Serum^19^					Limited specificity and PPV
CRP	84.6%	78.3%	36.7%	97.2%	
ESR	88.0%	65.1%	26.8%	97.4%	
Intraoperative histology^18^	73.7%	98.8%	94.1%	93.3%	Limited sensitivity

Abbreviations: NPV, Negative predictive value; PPV, Positive predictive value.

Tables 4 and 5 are provided are provided below.

With the addition of reference citations 16 to 20, the Reference section has been amended to correct reference 16 and add references 17 to 20. It should be captured as follows:

16. Higuera CA, Zmistowski B, Malcom T, Barsoum WK, Sporer SM, Mommsen P, et al. Synovial fluid cell count for diagnosis of chronic periprosthetic hip infection. *J Bone Joint Surg Am*. 2017; **99**(9): 753–759.

17. Rothenberg AC, Wilson AE, Hayes JP, O'Malley MJ, Klatt BA. Sonication of arthroplasty implants improves accuracy of periprosthetic joint infection cultures. *Clin Orthop Relat Res*. 2017; **475**(7): 1827–1836.

18. Kwiecien G, George J, Klika AK, Zhang Y, Bauer TW, Higuera Rueda CA. Intraoperative frozen section histology: matched for musculoskeletal infection society criteria. *J Arthroplasty*. 2017; **32**(1): 223–227.

19. Kuo FC, Lu YD, Wu CT, You HL, Lee GB, Lee MS. Comparison of molecular diagnosis with serum markers and synovial fluid analysis in patients with prosthetic joint infection. *Bone Joint J*. 2018; **100‐B**(10): 1345–1351.

20. Agten CA, Sutter R, Dora C, Pfirrmann CWA. MR imaging of soft tissue alterations after total hip arthroplasty: comparison of classic surgical approaches. *Eur Radiol*. 2017, **27**: 1312–1321.

We apologize for these errors.

